# Phylogeography of the common vampire bat (Desmodus rotundus): Marked population structure, Neotropical Pleistocene vicariance and incongruence between nuclear and mtDNA markers

**DOI:** 10.1186/1471-2148-9-294

**Published:** 2009-12-20

**Authors:** Felipe M Martins, Alan R Templeton, Ana CO Pavan, Beatriz C Kohlbach, João S Morgante

**Affiliations:** 1 Laboratório de Biologia Evolutiva e Conservação (LABEC), Instituto de Biociências - USP. Rua do Matão 277, Cidade Universitária, São Paulo - SP, CEP 05508-900, Brazil; 2 Washington University in St. Louis. One Brookings drive, campus Box 1137. St Louis, MO, 63130 USA

## Abstract

**Background:**

The common vampire bat *Desmodus rotundus *is an excellent model organism for studying ecological vicariance in the Neotropics due to its broad geographic range and its preference for forested areas as roosting sites. With the objective of testing for Pleistocene ecological vicariance, we sequenced a mitocondrial DNA (mtDNA) marker and two nuclear markers (RAG2 and DRB) to try to understand how Pleistocene glaciations affected the distribution of intraspecific lineages in this bat.

**Results:**

Five reciprocally monophyletic clades were evident in the mitochondrial gene tree, and in most cases with high bootstrap support: Central America (CA), Amazon and Cerrado (AMC), Pantanal (PAN), Northern Atlantic Forest (NAF) and Southern Atlantic Forest (SAF). The Atlantic forest clades formed a monophyletic clade with high bootstrap support, creating an east/west division for this species in South America. On the one hand, all coalescent and non-coalescent estimates point to a Pleistocene time of divergence between the clades. On the other hand, the nuclear markers showed extensive sharing of haplotypes between distant localities, a result compatible with male-biased gene flow. In order to test if the disparity between the mitochondrial and nuclear markers was due to the difference in mutation rate and effective size, we performed a coalescent simulation to examine the feasibility that, given the time of separation between the observed lineages, even with a gene flow rate close to zero, there would not be reciprocal monophyly for a neutral nuclear marker. We used the observed values of theta and an estimated mutation rate for the nuclear marker gene to perform 1000 iterations of the simulation. The results of this simulation were inconclusive: the number of iterations with and without reciprocal monophyly of one or more clades are similar.

**Conclusions:**

We therefore conclude that the pattern exhibited by the common vampire bat, with marked geographical structure for a mitochondrial marker and no phylogeographic structure for nuclear markers is compatible with a historical scenario of complete isolation of refuge-like populations during the Pleistocene. The results on demographic history on this species is compatible with the Carnaval-Moritz model of Pleistocene vicariance, with demographic expansions in the southern Atlantic forest.

## Background

The diversity of life in the Neotropics diversity has fascinated researchers worldwide. Many theories have been created to account for its extraordinary number of species, and these theories have been vigorously debated in the last decades [1]. One of the most cited and controversial is the Refuge Theory. Originally proposed by Haffer [2], it suggests that the Pleistocene glaciation cycles would create contraction and subsequent expansion of forested areas that in turn would create allopatry between populations of the same forest-dwelling species, leading to intraspecific differentiation and consequently speciation. Paleopalinogical studies demonstrate that the Neotropical forested areas have been very dynamic: the Atlantic forest and the Amazon were connected in the past [3,4], becoming separated as increasing aridity in the Tertiary triggered the formation of the belt of xeromorphic formations between them [5]. There seemed to be a predominance of arboreal vegetation during most of the Pleistocene with Amazonic and Atlantic forest tree species in areas that today lie in the dry diagonal that separates these two biomes [see [6,7]]. The extent to which these climatic fluctuations and associated vegetation changes affected the patterns of distribution and diversification of the fauna remains a central question in understanding the evolution of forest-associated taxa.

Species with broad geographical distribution in the Neotropics and a preference for forested habitats are specially suitable for the study of past fragmentation and connectivity between forested areas such as the Amazon and the Atlantic forest. The common vampire bat, *Desmodus rotundus *Geoffroy, 1810, is a species with a wide geographical distribution: it ranges from southern Mexico to northern Chile in the west, and the entire Brazilian and Uruguayan territory in the east [8]. Throughout its extensive range it occurs from sea level to over 3500 m of altitude and has been captured in habitats as diverse as rainforests and semi-arid vegetation. Due to the nature of previous phylogeographic studies in *D. rotundus*, it is believed that this species relies on either caves or forested areas for roosting, with the possibility of being captured in open sites while foraging [9]. Even with such a wide distribution, and with the existence of considerable morphological variation, there are no currently recognized subspecies for this taxon [8].

The common vampire bat feeds preferentially on medium to large sized mammals [8]. It lives in colonies that generally consist of fewer than 100 individuals. These colonies are formed by a dominant male, a number of peripheral males and several groups of unrelated or distantly related females [10-12]. The dominant male expels the juvenile males from its natal colony, and it has been suggested that the females are philopatric [11,12].

Previous studies that focused on the intraspecific genetic variation of the common vampire bat pointed to different phylogeographic scenarios. Baker et al. [13] analysed 22 allozyme loci in different colonies of *D. rotundus *bats in Central America and found low levels of genetic divergence both between individuals from the same and different colonies. Later, in a preliminary study carried in the Atlantic forest of Brazil, Ditchfield [14] analysed cytochrome b sequences from seven different individuals and found evidence for genetic structure together with sympatric haplotypes with high sequence divergence (4.0%). This value is much higher than the average divergence found for all other phyllostomid bats studied and suggets that, when studied on a larger scale, this bat could show phylogeographic structure for this marker.

Although largely used in phylogeographic studies, the mitochondrial DNA (mtDNA) is a single locus and bears limitations. Genealogies based on one single gene may be problematic because each reconstruction is just one point in the space of all possible genealogies. Depending on the genealogical history of the population studied, this approach may lead to erroneous inferences [15-19]. For example, loci under selection may deviate from the expected patterns of loci solely under demographic pressure and might mimic alternate demographic patterns [20]. Selection acts locally in the genome, while it is expected that demography affect all neutral loci uniformly [21].

Species that present high differentiation at the mtDNA level at times appear to be one single deme when nuclear loci are included in the analysis. That has been described for *Drosophila *[22], reptiles [23] and bats [24]. In the latter, the authors found population structure when analyzing distinct hibernation colonies of the bat *Myotis myotis *throughout the Alps using mtDNA, but no differentiation when microsatellites were used. The authors attributed the results to female philopatry and male-biased dispersal. A similar scenario was described for the ghost bat *Macroderma gigas *[25].

Given the factors explained above, this work aims at using mitochondrial and nuclear molecular markers to answer the following questions: (I) is there geographic structure for the common vampire bat *D. rotundus*? (II) Is this structure consistent across all markers? If not, what historical scenarios could be responsible for the incongruence? (III) Does the biogeographical pattern exhibited by the evolutionary lineages of this bat correspond to the expected distribution of lineages under the refuge hypothesis or any other theory created to account for the high diversity in the Neotropics?

## Methods

### Sampling, DNA extraction, amplification and sequencing

The Brazilian samples were collected by the authors under federal license issued by the Instituto Brasileiro do Meio Ambiente e dos Recursos Naturais - IBAMA (Brazilian Institute of Environment and Renewable Natural Resources) with 36 mm mistnets. The animals were sacrificed and all the samples are deposited in the Laboratório de Biologia Evolutiva e Conservação de vertebrados (LABEC) tissue bank. Samples from outside Brazil were donated by the American Museum of Natural History (AMNH), the Museum of Vertebrate Zoology (MVZ) and the Royal Ontario Museum (ROM).

Total DNA was obtained from ethanol-preserved liver or muscle and in two cases dried museum skin following Bruford et al. [26]. The protocol and primers for amplification of the cyt *b *fragments are described elsewhere [9]. The amplification and sequencing of the RAG2 gene followed the same protocol used for the mitochondrial gene. The primers used for amplification and sequencing of the RAG2 gene are described in Baker et al. [27]. The primers used for amplification and sequencing of the DRB intron 5 are described in Kupfermann et al. [28]. The eletropherograms were analyzed using the Sequence Navigator program and the Se-Al software [29] was used to align the DNA sequences by eye.

### Phasing of nuclear genotypes and recombination tests

Heterozygous nucleotide positions were identified by conspicuous double peaks in the electropherograms of both L and R strands. We used a Bayesian approach implemented in the software PHASE [30] to identify the haplotypes of heterozygous individuals.

In order to detect possible recombination events in the RAG2 and DRB markers, we used the RDP2 software [31]. This software applies six different methods for detecting recombination (see [32] and references therein). We used windows of 20, 50 and 100 bp in each of the scans.

### Phylogenetic analyses

Four different methods of phylogenetic inference were used. A maximum parsimony (MP) analysis was implemented, weighting all nucleotide changes and codon positions equally. Heuristic searches were run using random addition of taxa and tree bisection and reconnection algorithm (TBR), as implemented in the PAUP* software [32]. Bootstrap support [33] was estimated using 1000 replicates with heuristic mode parsimony. The maximum likelihood (ML) analysis used the GTR model of nucleotide substitution with different base frequencies and a gamma shape parameter of 0.3373, chosen using the Modeltest software [34]. The search for the best maximum likelihood tree also used the TBR algorithm. A distance-based analysis was implemented by using the substitution model described above to estimate distances and the neighbour joining algorithm to infer a phylogeny, also using the PAUP* software. Last, we used the Bayesian approach implemented in the Mr. Bayes software [35]. This method used the same substitution model as the ML analyses. The program ran 5 × 10^6 ^generations until the two chains had a standard deviation inferior to 0.01. The tree saved with posterior probabilities on the nodes was visualized using the Treeview software [36].

To complement the tree-based approaches we have also implemented Nested Clade Analysis (NCA) using the TCS software [37] with a statistical parsimony algorithm [38,39]. The haplotype network was used as input for the GEODIS software [40] and the result from this analysis was used on the software's inference key (release date: 11/11/2005).

### Population level and coalescent analyses

Analysis of molecular variance (AMOVA [41]) was used to quantify the extent of population subdivision using the Arlequin software [42]. Other summary statistics, as well as neutrality tests (carried over for testing the hypothesis of recent population expansion as expected under a Pleistocene refuge model) and dN/dS analyses were implemented using the DNAsp software [43].

To simultaneously estimate several population parameters, we used coalescent-based analyses. The following population parameters were estimated: time of separation between populations (t), θ (4Nμ), and a migration rate (m), when applicable. These analyses were implemented using the software MDIV [44] with a finite-sites model (HKY [45]) using the first 5 × 10^5 ^cycles as burn in and 5.5 × 10^6 ^total cycles. Different runs were carried out assuming either complete isolation between populations (m = 0) or populations exchanging migrants at a rate m (estimated by the software). In order to determine whether the model where m is estimated fits the data significantly better than the model of m = 0 we used the Akaike information criterion [46] to compare likelihoods, as described in Nielsen & Wakeley [44]. We compared the coalescent-based estimates of t and θ obtained to estimates using a methodology based on the net-sequence divergence measure (calculated using MEGA4 software [47]; see [48,49]) as described in Edwards & Beerli [50]. Two different mutation rates estimated for small-bodied mammals were used to calculate divergence times as applied by Hoffman et al. [51] for phyllostomid bats: 2.6% and 5.0% per million years. Generation time was assumed to be one year. We have also estimated a substitution rate for the RAG2 gene based on the data published by Baker et al. [27] on the phylogeny of the Phyllostomid family. We used Modeltest to estimate the model of nucleotide substitution and calculated the number of substitution using the origin of the family at 32 My ago as a calibration point as implemented by Teeling et al. [52]. The mutation rate was estimated at 1.94 × 10^-9 ^substitutions per site per year, similar to other mutation rates for nuclear genes in mammals [53].

### Coalescent simulations of nuclear sequence data

According to Hare [20], the average nuclear marker will not trace Pleistocene events in the same manner as mitochondral markers due to its larger effective size, lower mutation rates and diploid nature. If that statement is correct then the nuclear marker used in this study may be non-informative in phylogeographic analyses, even in the case of null or negligible gene flow between the demes. In order to examine the compatibility of a scenario where (I) there is null or negligible gene flow between two demes and (II) the mitochondrial analysis yields reciprocally monophyletic clades and the nuclear markers shows little to no phylogenetic signal, we decided to conduct a coalescent simulation of the nuclear data. We used the estimates of time of separation between the lineages and of mutation rates to generate DNA data for three populations. The next step is to use this data for generating phylogenetic trees and check for presence/absence of reciprocal monophyly of the populations. If the demographic scenario estimated for the observed data (with the given values of t and θ) generates no monophyly in ≥ 95% of the simulations, we can demonstrate that a pleistocenic scenario of complete isolation generates dissimilar results between mitochondrial and nuclear markers, as suggested by Hare [20].

We used two softwares that implement coalescent simulations to test this hypothesis. The first one is the SIMCOAL2 software [54]. This program allows for complex demographic models including population split, arbitrary migration rates and population contraction and expansion. The values used for this simulation were the ones estimated for the nuclear loci analyzed in this study: we used a θ value of 3.66, the mutation rate calculated for the RAG2 gene (1.94 × 10^-9 ^substitutions per site per year) and estimated the same number of chromosomes sampled for the actual data (24 for AMC, 50 for SAF and 62 for NAF). We ran 1000 iterations. We also used the MLCOALSIM software [55]. This coalescent simulator does not allow for very complex demographic settings: therefore we used an island model with negligible migration rates (m = 0.01), as null migration rates make the trees infinite and cause the program to crash. This simulation also used 1000 iterations. The outfiles generated by the software were converted to a format that was used as infile for the program TNT [56] using a LINUX script. The TNT program generated as outfile a matrix showing presence/absence of reciprocal monophyly for each of the simulated demes. On both simulations we used a biogeographic scenario based on the results obtained on the analyses implemented in this work: a split between the Amazon and the Atlantic forest around 900.000 YBP and a split between North and South Atlantic forest *circa *500.000 YBP.

## Results

### Molecular variation

For the mitochondrial marker, 118 individuals were sampled from 54 localities. A sequence of 832 bp of the cyt *b *gene was obtained for each specimen, and 72 haplotypes were described for *D. rotundus *and two for *Diphylla ecaudata *(used as outgroup). A total of 233 nucleotide sites were variable, and 152 of these variable sites were parsimony informative.

For the RAG2 gene 88 individuals were sequenced for a fragment 774 bp long, for a total of 176 chromosomes sampled. The individuals sampled belong to all the major localities sampled in the mitochondrial analysis except the Pantanal area. A total of 43 variable sites were identified, fifteen of which were heterozygous. After the analyses with the PHASE software, 45 unique haplotypes were identified. The haplotypic diversity for this dataset is very high (h = 0.896), but the nucleotide diversity was very low (π = 0.00314). The recombination analyses revealed no recombination events for this marker. For the DRB1 gene, only 62 sequences were obtained, each 282 bp long. Again, no samples from the Pantanal region could be amplified and sequenced. Fourteen variable sites were identified, including a two base pair indel at the end of this intron. The analysis with the software PHASE identified 18 unique haplotypes. Once again very low levels of nucleotide variability were observed (π = 0.00458). The table with all individuals, its respective localities, haplotypes and Genbank accession numbers can be found in the Additional file 1.

### Phylogenetic inference: mitochondrial marker

**Figure 1:**
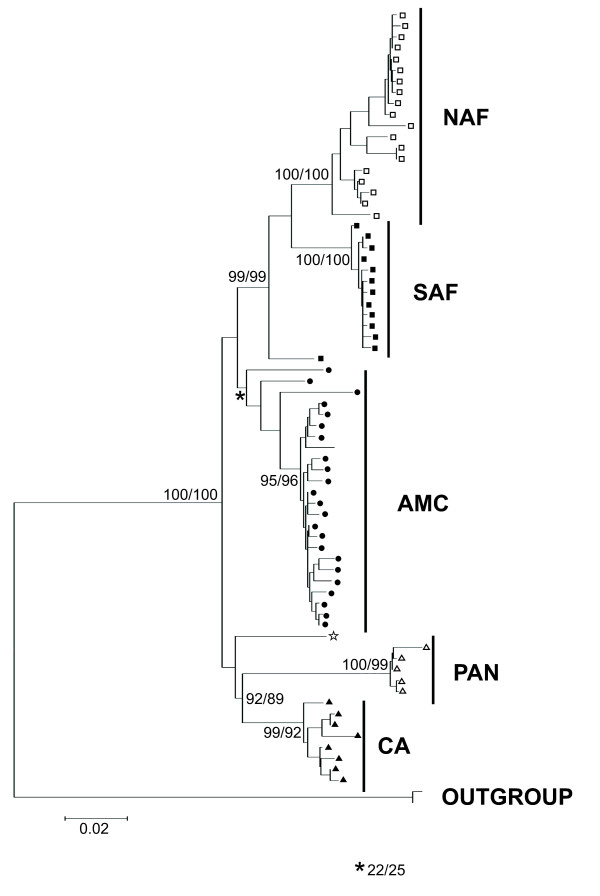
**Phylogenetic reconstruction of mtDNA haplotypes**. Maximum parsimony tree of all haplotypes identified. Posterior probabilities (left) and ML bootstrap supports (right) on critical nodes are shown. The major clades discussed in the text are highlighted at the rightmost of the tree. All phylogenetic reconstruction methods identified the five clades depicted in the figure. The clades are South Atlantic Forest (SAF), North Atlantic Forest (NAF), Amazon and Cerrado (AMC), Pantanal (PAN) and Central America (CA). The symbols at the terminal taxa represent the clade localities on Figure 2.

**Figure 2:**
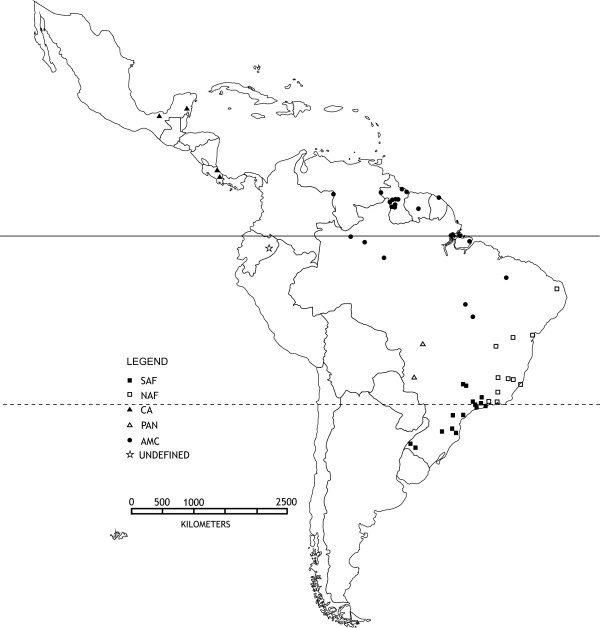
**Geographical representation of the mtDNA phylogenetic groups**. The geographical distribution of the five phylogenetic groups plotted on a map. The different geometric figures refer to localities sampled.

Figure 1 shows the MP phylogenetic reconstruction for the haplotypes described. The NJ phylogenetic tree was identical to the MP tree. The ML and bayesian analyses are largely congruent with this tree, with the exception of the relationships among a few terminal taxa. The figure shows that there are five major monophyletic clades representing distinct geographical regions and biomes. These are the following: Northern Atlantic Forest (NAF), Southern Atlantic Forest (SAF), Pantanal and Cerrado (PAN), Central America (CA) and Amazon and Cerrado (AMC). A single sample from Ecuador could not be assigned to any of the clades. Another sample from the southernmost area of the Atlantic forest is basal to the NAF and SAF clades. The distribution of each of these clades in the American continent can be seen in Figure 2. Although the clades had high bootsptrap and bayesian posterior probability support (except for the basal haplotypes on the AMC clade, which is discussed later), the relationship between the major clades - excluding the Atlantic forest monophyly - received very weak support and was not the same across all methods of phylogenetic reconstruction.

**Figure 3:**
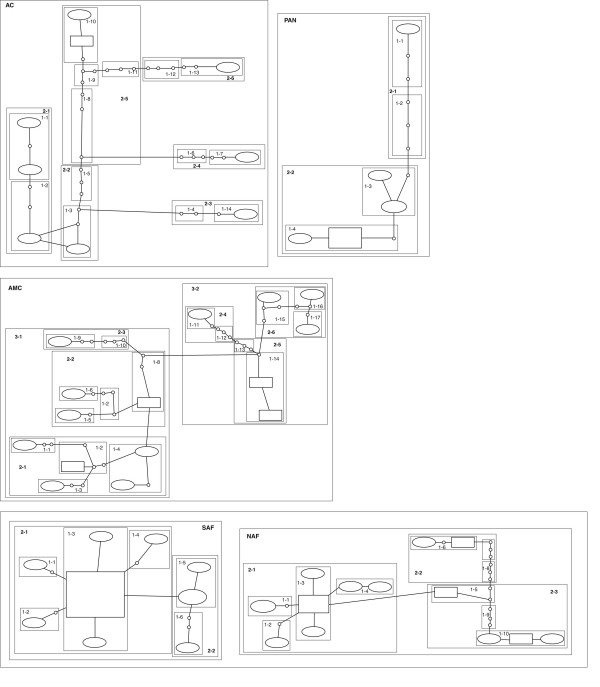
**mtDNA haplotype network**. Haplotype network of the mtDNA haplotypes generated by the software TCS and its hierarchical grouping. The last step clade is formed by the PAN, AMC and AC clade plus the (SAF+NAF) clade.

On the nested clade analysis, the TCS software identified the same clades as the tree-based methods. The high nucleotide divergence found between the clades made it impossible for the statistical parsimony algorithm to connect these networks even at 80% confidence intervals. The haplotype network is presented on Figure 3. To implement subsequent analyses using GEODIS, we decided to use the approach described in Durant et al. [57], where the last-step clade incorporates the clade formed by SAF and NAF together and the three clades (AMC, PAN and CA). This decision was made based on the fact that the relationship between these three clades is poorly resolved. Because the Atlantic forest was recognized as monophyletic in all analyses, we have clustered SAF and NAF in the same nesting clade for the NCA analyses as well. Both clusters were made independent on where and how these clades would connect. High and significant correlations between the haplotypes and their geographical localities were identified by the software GEODIS. The inference key identified the pattern observed to the Atlantic forest as a whole as allopatric fragmentation. In the case of the last step clade, our sampling does not allow to discriminate between allopatric fragmentation and isolation by distance - which was predictable, given the large gaps in sampling, especially in central Brazil and northwestern South America.

### Phylogenetic inference: nuclear markers

With the extensive sharing of haplotypes and low nucleotide variability observed for the nuclear markers, all tree-based methods were non-informative. We therefore decided to implement unrooted networks to extract more information from the data. For the RAG2 gene, the analysis with the TCS software generated a very large number of ambiguous connections. In order to carry the subsequent analyses, single-copy non-internal haplotypes with multiple connections were removed from the data matrix. The final data matrix generated the network used as input in the GEODIS software. The network for the DRB intron used all haplotypes. The hierarchical grouping of clades was done manually. The analyses carried with the GEODIS software found no statistical correlation between clades and geography for both markers, meaning that all analyses were inconclusive.

### Population-level analyses and coalescent estimates

The structure found in the phylogenetic analyses for the mitochondrial marker was also observed in the population-level analyses. The overall fixation index was high and significant (Fst = 0.16; p << 0.05). All pairwise comparison between the clades identified were also significant. The AMOVA result reflect the same pattern: 84.15% of the variance is found within populations. All neutrality tests applied were non-significant except for the SAF clade (Tajima's D = -2.18; p = 0.003; Fu's Fs = -4.72; p = 0.002). The time divergence estimates for these clades are in Table 1. In all but two pairwise comparisons (SAF × PAN and NAF × PAN) the best fitting model was the one of complete isolation (m = 0). This result yields flat likelihood surfaces for the parameter t making it impossible to calculate coalescent time divergence estimates. The coalescent and non-coalescent time estimates for each comparison are very similar and all within the Pleistocene epoch.

**Table 1:** Pairwise divergence times for the mtDNA clades described.

	Time estimate (coalescent)	Time estimate (non-coalescent)
**SAF × NAF**	400,000-800,000	350,000-700,000 y

**SAF × AMC**	535,000-1 My	390,000-800,000 y

**SAF × PAN**	-	835,000-1,6 My

**NAF × AMC**	540,000-1,1 My	340,000-680,000 y

**NAF × PAN**	-	770,000-1,5 My

**AMC × PAN**	640,000-1,2 My	545,000-1.1 My

**CA × SAF**	582,000-1,1 My	520,000-1 My

**CA × NAF**	600,000-1,3 My	520,000-1 My

**CA × AMC**	600,000-1,3 My	360,000-750,000 y

**CA × PAN**	685-1,35 My	770,000-1,6 My

Coalescent and non-coalescent divergence time estimates between the mitochondrial clades described in the phylogenetic analyses.

For the RAG2 gene, the mean nucleotide divergence in the sample was very low (0.6%) and there is an extensive sharing of haplotypes between very distant localities, including the localities belonging to distinct mitochondrial lineages. At the same time, there is an equal number of exclusive haplotypes for each of these regions.

Given the results obtained for the mtDNA, we decided to conduct further analyses utilizing the mtDNA clades as populations for the nuclear markers. Even with this extensive sharing of haplotypes, Fst values between the locations demarcated by the mitochondrial clades were all significant for both markers. The AMOVA analysis showed that 95% of the allele variance is found within populations for RAG2, and 92.35% for DRB. All neutrality tests were non significant for RAG2 except the SAF (D = -1.52, p = 0.042; Fs = 8.68, p = 0.002), which is identical to the result obtained for mtDNA. This result could not be replicated by the DRB intron, probably due to poor sampling for these localities (only ten individuals). Table 2 summarizes the population genetics results for the RAG2 marker.

**Table 2:** Population genetics analysis results for the nuclear marker RAG2.

	SAF	NAF	AMC
**SAF (20)**	-	0.018	0.036
**NAF (19)**	6	-	0.009
**AMC (17)**	5	5	-

The table shows the pairwise Fst values for the populations in the above diagonal; below diagonal, the number of shared alleles between each pair of populations. The number of total alleles for each population is in bold after the population name.

The results obtained for dN/dS analyses for the nuclear markers showed approximately ten synonymous substitutions for each non-synonymous, congruent with the expected pattern for neutral markers described in the literature [58].

The coalescent analyses using the MDIV software was implemented in the RAG2 dataset and yielded a time of divergence of 500,000 years (95% confidence interval 229,000-940,000 years) between the two Atlantic forest clades and 900,000 years (95% confidence interval 524,000-1,400,000 years) between the Atlantic forest clades and the AMC clade, values congruent with the ones obtained for the mitochondrial marker and that were used in the coalescent simulations. We decided to not implement the coalescent analyses on the DRB intron due to a lack of reliable mutation rate estimates.

### Coalescent simulations

The simulations generated by the sofware SIMCOAL yield unrealistic results. The outfiles were run in the Arlequin software and in all the outfiles generated the number of variable sites was equal to the number of nucleotide sites. Given these conditions, each chromosome was a distinct haplotype. These results are completely incongruent with the observed data and had to be discarded from the analyses.

We decided to concentrate our efforts on the MLCOALSIM software. The outfiles generated had similar haplotype diversity (average 0.94; s.d. 0.024) and number of segregating sites (average 33; s.d. 1.41) values between the demes in average similar to the observed data for RAG2. Given these conditions we ran the 1000 iterations on the TNT software using maximum parsimony. The results of these analyses are summarized in Table 3. The results show that there is no clear distinction between all possible scenarios involving reciprocal monophyly of the clades, i.e., all possible scenarios have similar probabilities of ocurring given the history and demographic parameters observed in the data and used (with adaptations) in the simulation. Therefore, no scenarios could be confirmed or excluded.

**Table 3:** Coalescent simulations and clade monophyly.

	*No reciprocal monophyly*	*Reciprocal monophyly for SAF*	*Reciprocal monophyly for NAF*	*Reciprocal monophyly for AMC*	*Reciprocal monophyly for the three clades*
**Frequency**	18%	54.2%	52.3%	55%	31.7%

Results of the coalescent simulation analyses showing the relative frequencies of each outcome regarding monophyly of the clades.

## Discussion

### Biogeographic pattern

It comes as no surprise that species with wide geographical distributions consist of two or more evolutionary units when molecular markers are studied. In the case of the common vampire bat, the population subdivision found for the mitochondrial marker does not translate into colonies or hibernation sites, as has been described for other bats (see [59] and [24]). The subdivision corresponds to different ecodomains and has correspondence in other vertebrate organisms. Therefore we are dealing with a complex pattern that needs to be carefully discussed.

The common vampire bat can fly 20 km from roost to feeding site in a single night [60]. It inhabits all the biomes that exist in the Neotropics, from seasonally flooded forests to semi-arid environments, and from sea level to 3600 meters of altitude. Therefore there are no identifiable physical barriers for dispersal and gene flow in the distributional range of this bat. In this case, it is likely that ecological barriers serve as a possible explanation to the structure found for this bat. All the divergence times estimated using coalescent and non-coalescent approaches fall within the Pleistocene epoch, suggesting that this bat is indeed susceptible to forest fragmentation.

The east/west separation detected by the mitochondrial marker in *D. rotundus *is very clear and coincides with the biogeographic provinces described by Koopman [61] for South America, but unfortunately, when using this marker, the relationship between the western clades remains obscure. On the one hand, the high nucleotide divergence to the outgroups of the analysis (up to 30%) may have influenced the topology of the tree. On the other hand the lack of resolution regarding the phylogenetic relationships between these three clades may reflect concomitant historical events of divergence. In this case, there would be an interesting corridor for gene flow along the eastern slopes of the Andes cordillera, that generated the clade formed by PAN and CA that showed high bootstrap support as shown in Figure 1. The other ancestral clade would be formed along the Guiana and Brazilian shields (that originated the AMC and the Atlantic forest clades).

The high levels of sequence divergence may also be responsible for low bootstrap values showed by the AMC clade; but since this clade was consistently present with the exact same topology in all methods employed in this study, we consider this clade to be reflecting the true relationship between the sampled haplotypes. Since two of the three basal haplotypes are peripheral in the clades geographical distribution (one in Venezuela and one in Marajó Island, at the mouth of the Amazon river), it is possible that fine-scale sampling could reveal a better resolution of this very large geographical area.

The Atlantic forest has become separated of the other area clades during the Pleistocene epoch, a result that is congruent with the appearance of a dry belt separating this forested area from the Amazon. This is the first study to suggest a Pleistocene separation of the Atlantic forest and the Amazon based on the phylogeographic data collected on a vertebrate species using molecular-based estimates of divergence times.

This work describes the Atlantic forest of Brazil as a composite area, with northern and southern components. The latitudinal division of this area has been recognized using parsimonious analysis of endemicity in amphibians [62,63], reptiles [64], birds [65] and harvestmen [66]. More recently, phylogeographic studies that used mtDNA described this structure in organisms as diverse as birds [67], pit vipers [68], non-volant small mammals [69], canids [70] and the bat species *Carollia perspicillata *[14]. All the studies that implemented time estimates yielded Pleistocene divergence times for this event. There are numerous paleopalinological and sediment studies that describe that this region has been fragmented with dry open areas related to glaciation-driven events during the Pleistocene [71,72,73,74].

In a recent study, Carnaval and Moritz [75] generated climatic simulation data and cross-referenced their results with phylogeographic and paleopalinological studies. The authors describe a scenario where in its northern portion the Atlantic forest have always supported an evergreen forest even during the driest conditions, while south of the Doce river the climatic conditions would not support a forest formation. The authors suggest that in its current southern distribution, the Atlantic forest was probably fragmented in several small patches in the wettest areas, a scenario that was proposed before by Whitmore and Prance [76]. The results shown here are congruent with this scenario: the SAF clade is the only one with significant evidence of population expansion for two different markers. According to Lessa et al. [77], refuges would not only create geographic structure that cannot be associated with conspicuous contemporaneous physical barriers on molecular markers, but these same markers should bear the footprints of a population expansion related to the end of the last glacial cycle. The results obtained here reflect the predictions of Pleistocene forest dynamics: the time divergence estimates all fall within this epoch and the estimated historical demography is congruent with refugia.

The existence of a Atlantic forest lineage that is basal to both SAF and NAF comes as a surprise, specially because this lineage is at the southernmost end of the Atlantic forest distribution - where according to the Carnaval-Moritz model, there should be no forests at the last glacial maximum. Two different scenarios can be hypothesized for this data: (I) retention of ancestral polimorphism associated with demographic expansion [78] from the hypothesized São Paulo refugium [9,75] or (II) there could be more geographic structure regarding the common vampire bat that the sampling presented here allows discriminating. This particular haplotype could represent another lineage and a possible contact zone between this lineage and the SAF.

### Mitochondrial and nuclear incongruence

The incongruence between the results observed for the mitochondrial and nuclear markers could be due to two different scenarios: complete fragmentation and incongruence between markers due to the nature and characteristics of each molecular marker or long-term female philopatry and male-biased dispersal. We will discuss each of these hypotheses in detail.

The first possibility - of complete isolation between populations but no footprints in nuclear markers due to larger effective size and lower mutation rates - was the reason behind the coalescent simulations carried in this study. The results of the simulations have shown that given the mutation rate, time of separation and effective population size calculated for these bats, an average nuclear marker might or might not reflect the true demographic history of this species with similar probabilities. Given that, we believe that the best way to approach the question on whether the structure found is valid or the outcome of long-term female philopatry would be to sequence a larger number of loci - at least 16 according to Moore [79] - in search of more accurate phylogenetic reconstructions or use other nuclear markers such as the Y chromosome or microsatellites, all of which are beyond the scope of the present analysis. The RAG2 marker was chosen based on the work by Lewis-Oritt et al. [80] that described relatively high levels of intraspecific divergence for this marker in Moormopidae bats (comparable in some cases to mtDNA intraspecific divergence in bats). The DRB intron was chosen due to the possibility of studying balancing selection on the common vampire bat - as described for many of the genes that comprise the major histocompatibility complex (MHC) in vertebrates. This work shows that these two markers do not seem appropriate for intraspecific phylogeographic studies.

Females of *D. rotundus *usually remain in their natal colonies after they reach maturity, as observed by Wilkinson [11], but they may also join a new group not far from their birthplace. In addition, females do occasionally migrate among roosts, so there is evidence of some adult female dispersal. Under these conditions of female philopatry with restricted dispersal, the expected pattern of differentiation would be one of isolation by distance [9]. However, in our analyses we find low sequence divergence among localities at large geographic distances (over 400 km), and large increases in genetic differentiation taking place over relatively short distances. This pattern is particularly clear in the analysis of the Atlantic forest. The NAF and SAF possess similar geographical area and distances between the localities sampled. Both clades show low differentiation (maximum haplotype divergence being under 1% in SAF and under 3% in NAF) over a broad geographical range (see Figure 2), but are highly divergent from one another (mean divergence 6.6%), with haplotypes differing by 7% separated by less than 200 km. The NCA results point towards the same direction in opposition to isolation by distance. We believe that if the sampling gaps were filled, this outcome on the inference key (allopatric fragmentation) would be observed repeatedly among all clades. The results observed for the mtDNA marker show strong evidence of historical fragmentation, even with limited information on the extent of historical and current male-mediated gene flow.

## Conclusions

The phylogeographic pattern described for the common vampire bat *Desmodus rotundus *is characterized by Pleistocene ecological vicariance. The mitochondrial marker showed deep divergence between reciprocally monophyletic clades representing distinct ecodomains within the Neotropics. There was also a clear East/West division within South America, where the coastal Atlantic forest was separated from the Amazon and the Pantanal by the Brazilian dry diagonal of open formations. The times of separation between the lineages are all within the Pleistocene epoch. The phylogenetic pattern is congruent with many other Neotropical clades. In addition, the historical demography, with a population expansion at the southern end of the distribution, is compatible with the Carnaval-Moritz model of historic Atlantic forest dynamics. The coalescent simulations showed that, given the population parameters estimated for this species, a nuclear marker may or may not recover Pleistocene population history with similar probabilities.

The next step in revealing the true nature of the interactions between the mitochondrial clades indentified and the species status within *D. rotundus *will be finding the exact locations of the contact zones between these clades and studying in detail the different kinds of interactions between the animals in these localities. This study would consider not only a multiloci approach but also field observations on ecology and behavior. We are also on the way to conduct experiments with bats genotyped for the different clades under controlled laboratory conditions, to test for reproductive isolation and the possible ramification of the hybridization between the animals belonging to different clades.

## Authors' contributions

FMM carried out the research, carried out and interpreted the analyses and wrote the manuscript. AT co-supervised the project, interpreted the analysis and revised the manuscript. ACOP and BCK carried out laboratory experiments and aligned part of the sequences. JSM supervised the project and revised the manuscript. All authors participated in the discussions and approved the final manuscript.

## Supplementary Material

Additional file 1 **Samples used in this study with Genbank accession numbers**. Collection of localities sampled with the number of individuals sampled (all in Brazil, except when indicated in bold) with its respective Genbank Access number for each of the markers used. Latitudes and longitudes are in decimal points. Click here for file
