# Supplementary material for: Phylogeography of the common vampire bat (Desmodus rotundus): Marked population structure, Neotropical Pleistocene vicariance and incongruence between nuclear and mtDNA markers
Source: BMC Evol Biol. 2009 Dec 20;9:294. doi: 10.1186/1471-2148-9-294 (PMC2801518; doi:10.1186/1471-2148-9-294)
Supplement: Additional file 1 — Samples used in this study with Genbank accession numbers. Collection of localities sampled with the number of individuals sampled (all in Brazil, except when indicated in bold) with its respective Genbank Access number for each of the markers used. Latitudes and longitudes are in decimal points. [file 1471-2148-9-294-S1.DOC]

| N | Locality | Clade | LAT | LONG | mtDNA Genbank number | RAG2 Genbank number | DRB intron5 Genbank number |
| --- | --- | --- | --- | --- | --- | --- | --- |
| 4 | Ibitipoca (MG) | NAF | -21.71 | -43.91 | FJ847446 FJ847455 FJ847456 | FJ863172-7 | - |
| 2 | Cajuru (SP) | SAF | -2.6 | -48.3 | FJ847447 FJ847460 | FJ863262-3 | - |
| 2 | Jundiaí (SP | SAF | -23.18 | -46.86 | FJ847461 FJ847462 | FJ863182-3 FJ863188-9 | - |
| 2 | Valença (BA) | NAF | -13.39 | -39.08 | FJ847452 FJ847453 | FJ863164-7 | - |
| 1 | Ilhabela (SP) | SAF | -23.78 | -45.35 | FJ847447 | FJ863180-1 | - |
| 3 | Intervales (SP) | SAF | -23.6 | -46.61 | FJ847459 | FJ863186-7 | - |
| 2 | Caratinga (MG) | NAF | -19.78 | -42.13 | FJ847448 FJ847454 | FJ863168-71 | - |
| 1 | Salesópolis (SP) | SAF | -23.53 | -45.83 | FJ847447 | FJ863266-7 | - |
| 1 | Cunha (SP) | NAF | -23.08 | -45 | FJ847463 | FJ863184-5 | - |
| 8 | Ribeirão Pires (SP) | SAF | -23.71 | -46.41 | FJ847447 FJ847461 FJ847464 FJ847465 | FJ863132-47 | - |
| 1 | Sales de Oliveira (SP) | SAF | -20.76 | -47.85 | FJ847447 | - | - |
| 2 | Santa Isabel do Rio Negro (AM) | AMC | -0.4 | -65.033 | FJ847449 FJ847450 | FJ863202-3 | GQ866047 |
| 1 | Barcelos (AM) | AMC | -1 | -62.96 | FJ847451 | FJ863162-3 | - |
| 8 | Paraisópolis (MG) | SAF | -22.55 | -45.78 | FJ847447 FJ847457 FJ847458 | - | - |
| 1 | Salvaterra (PA) | AMC | -0.76 | -48.5 | FJ847475 | FJ863178-9 | - |
| 10 | Miranda (MS) | PAN | -20.23 | -56.33 | FJ847470 FJ847471 FJ847472 FJ847473 FJ847474 | - | - |
| 5 | Bananal (SP) | NAF | -22.68 | -44.31 | FJ847455 FJ847479 FJ847480 | FJ863148-55 | - |
| 3 | Lençóis (BA) | NAF | -13.81 | -41.71 | FJ847476 FJ847477 FJ847478 | FJ863156-61 | - |
| 13 | Bocaiúva do Sul (PR) | SAF | -25.18 | -49.13 | FJ847447 FJ847462 FJ847465 FJ847505 FJ847506 | FJ863244-61 | GQ866026-30 |
| 15 | Castelos (ES) | NAF | -20.6 | -41.2 | FJ847448 FJ847456 FJ847476 FJ847503 FJ847504 FJ847505 | FJ863214-43 | GQ866050-65 |
| 2 | Pedro Leopoldo (MG) | NAF | -19.63 | -44.05 | FJ847481 | - | - |
| 2 | Marliéria (MG) | NAF | -19.71 | -42.75 | FJ847454 | - | - |
| 1 | Itacarambi (MG) | NAF | -15.08 | -44.11 | FJ847482 | FJ863264-5 | - |
| 3 | Cananéia (SP) | SAF | -25 | -47.95 | FJ847447 FJ847506 | FJ863198-201 | - |
| 1 | Araruna (PB) | NAF | -6.53 | -35.73 | FJ847514 | FJ863282 | - |
| 4 | Jangada (MT) | PAN | -15.27 | -55.23 | FJ847470 | - | - |
| 22 | Ilha das Cavianas (PA) | AMC | -0.15 | -50 | FJ847509 | - | GQ866048 |
| 1 | Emas (GO) | AMC | -15.63 | -47.82 | FJ847469 |  | - |
| 1 | Porto Nacional (TO) | AMC | -11 | -48 | FJ847483 | - | GQ866038 |
| 4 | Dois irmãos (TO) | AMC | -9.21 | -48.95 | FJ847483 | FJ863190-5 FJ863268-9 | GQ866041 |
| 1 | Estreito (MA) | AMC | -5.18 | -43.25 | FJ847484 | FJ863196-7 | GQ866039-40 |
| 5 | Alegrete (RS) | SAF | -29.76 | -55.76 | FJ847447 FJ847510 FJ847511 | - | GQ866024-25 |
| 3 | Itaqui(RS) | SAF | -29.13 | -56.55 | FJ847447 FJ847512 | - | - |
| 3 | Gover nador Celso Ramos (SC) | SAF | -27.6 | -51.4 | FJ847447 | - | - |
| 1 | Porto Belo (SC) | SAF | -27.15 | -49.53 | FJ847447 | - | - |
| 1 | Rancho Queimado (SC) | SAF | -27.68 | -49 | FJ847447 | - | - |
| 1 | Manaus (AM) | AMC | -3.11 | -60.025 | FJ847508 | - | GQ866031-32 |
| 1 | Rio Anotaie Tucumaque (AP) | AMC | 0.035 | -51 | FJ847513 | - | GQ866033 |
| 1 | Rio Anacuí Tucumaque (AP) | AMC | 0.06 | -51.01 | FJ847516 | - | - |
| 12 | APA do Curiaú (AP) | AMC | 0.13 | -51.06 | FJ847515 FJ847516 FJ847517 | FJ863274-9 | GQ866033 GQ866043-46 |
| 4 | Cano Palmo biological station, Costa Rica | CA | 10.58 | -83.15 | FJ847486 FJ847501 FJ847502 | FJ863281 | - |
| 2 | Prov. Limon, Costa Rica | CA | 9.51 | -83.15 | FJ847467 FJ847468 | FJ863204-7 | - |
| 1 | Parque nacional Montecristo, El Salvador | CA | 13.86 | -89.58 | FJ847497 | FJ863273 | GQ866042 |
| 1 | Paracou, French Guiana | AMC | 5.37 | -52.88 | FJ847466 | - | - |
| 1 | Namai Creek, Guiana | AMC | 5.8 | -61.05 | FJ847487 | FJ863210-11 | - |
| 1 | CEIBA biological station, Guiana | AMC | 6.51 | -58.16 | FJ847491 | - | - |
| 1 | Mango Landing, Guiana | AMC | 6.25 | -57.51 | FJ847487 | - | - |
| 1 | Shanklands, Guiana | AMC | 5 | -59 | FJ847491 | - | - |
| 1 | Kabulakalli Landing, Guiana | AMC | 4.5 | -59.8 | FJ847491 | - | - |
| 1 | Mount Ayanganna, Guiana | AMC | 4.91 | -49.45 | FJ847487 | FJ863285-6 | - |
| 1 | Sand Stone, Guiana | AMC | 4.5 | -59 | FJ847487 | - | - |
| 1 | Tropembos, Guiana | AMC | 5 | -58.66 | FJ847491 | FJ863212-3 | - |
| 1 | Pobawau Creek Mouth, Guiana | AMC | 9.6 | -48 | FJ847487 | - | - |
| 1 | Surama, Guiana | AMC | 4.16 | -59.08 | FJ847487 | - | - |
| 1 | Kanuku Mountain, Guiana | AMC | 3.85 | -59.6 | FJ847491 | - | - |
| 2 | Quarter Mile Landing, Guiana | AMC | 3.95 | -59.13 | FJ847491 | - | - |
| 1 | Pobawau Creek Mouth, Guiana | AMC | 3.26 | -58.25 | - | FJ863208-9 | - |
| 2 | Laguna Noh-Bec, Mexico | CA | 19.08 | -88.18 | FJ847489 | FJ863271-2 | - |
| 3 | Jonuta, Mexico | CA | 18.08 | -92.13 | FJ847488 FJ847489 | FJ863280 | - |
| 1 | Bakhuis, Surinam | AMC | 3.8 | -55.7 | FJ847493 | FJ863283-4 | - |
| 1 | Pompeya Sur, Yasuni National Park, Ecuador | - | -1 | -77 | FJ847511 | - | GQ866036 GQ866037 |
| 1 | Puerto Ayacucho, Venezuela | AMC | 5.66 | -67.62 | FJ847490 | - | GQ866035 |
| 1 | San Cristobal Acasaguastlan, Guatemala | CA | 14.91 | -89.88 | - | FJ863270 | - |
